# Cognitive behaviour therapy for chronic fatigue syndrome: Authors' reply, naturalistic outcomes paper

**DOI:** 10.1177/01410768211017775

**Published:** 2021-05-27

**Authors:** T Chalder, J Adamson, A Santhouse, S Wessely

**Affiliations:** Department of Psychological Medicine, King’s College London, UK

We previously reported on routine clinical outcomes after cognitive behaviour therapy for chronic fatigue syndrome in an NHS clinic.^1^ We found that fatigue, physical functioning and social adjustment all significantly improved, providing some evidence that results from randomised controlled trials can be extrapolated to everyday clinical settings.

Vink, Vink-Niese^2^ and Tack^3^ raised a number of issues with our paper, which we will respond to. Although the NHS clinic sees patients with both chronic fatigue and chronic fatigue syndrome, all patients included in this evaluation met NICE criteria for chronic fatigue syndrome and were assessed by an experienced clinician prior to treatment.

A proportion of scores on the SF-36 physical functioning scale were missing. This was not related to dropout but due to the measure being introduced two years after the other routine outcome data collection had started. However, the sample size was reasonable. We included this measure as it is routinely used in trials of behavioural treatments for chronic fatigue syndrome.

The amount of cognitive behaviour therapy offered was flexible depending on patient need. Those with missing data did not all drop out of therapy. We defined dropout as those who did not complete any measures at discharge and follow-up at three months. As a naturalistic study, we felt it was important to include as many participants as possible. With this in mind, we also chose a statistical approach that manages missing data. Furthermore, we conducted a dropout analysis and were clear about this being a limitation. We acknowledged in the paper that dropouts were more ill at the start. However, this does not detract from the fact that many of those who adhered to the full course of cognitive behaviour therapy for chronic fatigue syndrome saw significant improvements. The fact that improvement occurred for a high percentage of people who completed treatment is a useful observation for patients and clinicians alike.

We do not feel that the use of ‘subjective’ as opposed to ‘objective’ measures is a weakness. Chronic fatigue syndrome remains defined by subjective criteria – namely symptoms, and no ‘objective’ biomarker has been found to date. Even when that happens, we continue to expect that patient-reported outcome measures will remain as important if not more important than objective measures. In the end, clinicians will continue to find there is no substitute to listening to the patient when deciding the success or otherwise of management.

Patients were largely satisfied with cognitive behaviour therapy, with over 90% rating their satisfaction as at least slightly satisfied and 45% as very satisfied. These figures represent all patients who completed self-report measures at discharge and are therefore commensurate with all other reported figures at discharge. Although we did not report patient satisfaction at the follow-up, satisfaction rates remained consistent with rates at discharge.

In conclusion, we disagree with the conclusions of Vink, Vink-Niese and Tack. While some patients do remain disabled, significant improvements with medium effect sizes in self-reported measures is a positive outcome for a large number of patients who are seen in a specialist clinic in the UK.
